# Bioethical and critical consciousness in clinical translational neuroscience

**DOI:** 10.1017/cts.2025.5

**Published:** 2025-01-22

**Authors:** Angela Fang, Riana Elyse Anderson, Sierra Carter, Kristen Eckstrand, Kean J. Hsu, Shawn Jones, Maria Kryza-Lacombe, Andrew Peckham, Greg J. Siegle, Lucina Q. Uddin, Mariann Weierich, Mary L. Woody, Judy Illes

**Affiliations:** 1 University of Washington, Seattle, WA, USA; 2 Columbia University, New York, NY, USA; 3 Georgia State University, Atlanta, GA, USA; 4 University of Pittsburgh, Pittsburgh, PA, USA; 5 National University of Singapore, Singapore; 6 Virginia Commonwealth University, Richmond, VA, USA; 7 Veterans Affairs Mental Illness Research Education and Clinical Centers, San Francisco, CA, USA; 8 University of California San Francisco, San Francisco, CA, USA; 9 U.S Department of Veterans Affairs, University of Massachusetts Chan Medical School, Worcester, MA, USA; 10 University of Pittsburgh School of Medicine, Pittsburgh, PA, USA; 11 University of California Los Angeles, Los Angeles, CA, USA; 12 University of Nevada, Reno, NV, USA; 13 University of British Columbia, Vancouver, BC, Canada

**Keywords:** Bioethics, translational, neuroscience, mental health disparities, mental health disorders

## Abstract

Clinical translational neuroscience (CTN) is positioned to generate novel discoveries for advancing treatments for mental health disorders, but it is held back today by the siloing of bioethical considerations from critical consciousness. In this article, we suggest that bioethical and critical consciousness can be paired to intersect with structures of power within which science and clinical practice are conducted. We examine barriers to the adoption of neuroscience findings in mental health from this perspective, especially in the context of current collective attention to widespread disparities in the access to and outcomes of mental health services, lack of representation of marginalized populations in the relevant sectors of the workforce, and the importance of knowledge that draws upon multicultural perspectives. We provide 10 actionable solutions to confront these barriers in CTN research, as informed by existing frameworks such as structural competency, adaptive calibration models, and community-based participatory research. By integrating critical consciousness with bioethical considerations, we believe that practitioners will be better positioned to benefit from cutting-edge research in the biological and social sciences than in the past, alert to biases and equipped to mitigate them, and poised to shepherd in a robust generation of future translational therapies and practitioners.

## Introduction

Clinical translational neuroscience (CTN), sometimes also referred to as Mental Health Science [1], is an emerging trajectory within the broader field of mental health care delivery that focuses on understanding biological contributions to behavior and behavior change. Its goal is to advance therapeutic benefit for people with mental health and related conditions. Modern treatments have not benefited fully from contemporary scientific advancements or collaborations between clinicians and neuroscientists [1]. CTN has brought together diverse theories and methods across subfields of neuroscience, involving the cognitive, biological, affective, and social bases of behavior. Like any science, however, CTN is vulnerable to biases held by the individuals and systems contributing to it.

For clinical and research scientists in the community of readers of this journal and others related to mental health care research and delivery, these issues are particularly salient in terms of understanding how bias impacts translational efforts across diverse patient populations and clinical settings and how it affects the translational workforce. We suggest that when paired with bioethical consciousness, critical consciousness can uplift and support an integrative understanding of both the principles that guide health care, including decisions for mental health, and intersections with power structures within which science and clinical practice are conducted. Among bioethical frameworks, Beauchamp and Childress’ four principles of respect for autonomy, nonmaleficence, beneficence, and justice [2] are well known and guide the translation of CTN findings into practice and care. Without attention to critical consciousness alongside it, however, we argue that this classic framework, like others that are similarly comprehensive and robust (e.g. [3,4]), cannot adequately embody laws and policies within clinical systems that could mitigate contemporary inequities. The downstream consequence is that the full and significant opportunity for CTN research to advance mental health intervention and service delivery research may be hampered.

By bringing critical consciousness to the foreground and urging its complementarity with bioethical consciousness, clinical translational neuroscientists may enhance translational efforts by (1) advancing the measurement, interpretation, and reconciliation of biological variables with psychological and social ones [5,6]; (2) reforming practices in research and clinical settings that have historically excluded marginalized communities [7]; and (3) promoting the training of scientists and clinicians in the connections between person-level and institution-level racism based on diversity science [8] and the structural determinants of health [9].

Insights from CTN have already entered mental health clinics through popular knowledge of pharmacologics (e.g., cannabis, ketamine, psychedelics), brain stimulation techniques (e.g., transcranial magnetic stimulation), and functional magnetic resonance imaging (fMRI). Advancements in understanding cellular, molecular, and circuit mechanisms of various neuromodulators in psychiatric disorders have opened the door to new neuroscience-informed clinical taxonomies [10] and new classes of neuroscience-informed interventions, which mechanistically target dysfunctional circuits and pathologies [11]. However, without an integrated bioethical and critical consciousness lens, knowledge of such translational discoveries may be limited to subsets of the population and promulgate disparities in the sociodemographic representation of patients, stigma, and health outcomes. Restricted access to interventions and services, for example, in studies of neurocognitive development and functioning, is naturally an undesirable outcome. [12,13].

Our primary objectives here are to (1) evaluate the current state of evidence on barriers to why neuroscience has not penetrated mental health clinics from a critical consciousness lens, (2) better serve diverse and marginalized populations using existing frameworks that may address these barriers, and (3) propose some solutions for promoting bioethical and critical consciousness in CTN research.

## Barriers to adopting neuroscience in mental health clinics

To quote the scholar, author, and activist, Angela Davis (2015) [14], “I feel that if we don’t take seriously the ways in which racism is embedded in structures of institutions, if we assume that there must be an identifiable racist who is the perpetrator, then we won’t ever succeed in eradicating racism.” Racist practices are embedded in life experiences, in research, and in systems that produce research. Below, looking through the lens of critical consciousness, we describe major barriers to the adoption of neuroscience research in mental health clinics from a critical lens: a historical consciousness in society and in research that has been dismissive of racism; problematic methods, practices, and norms; and mismatch with clinician needs.

### Historical consciousness

Academia has a tradition of sharing certain characteristics of white supremacy culture, such as objectivity and paternalism [15]. Scientific objectivity may manifest as being indifferent to the life experiences of research participants. For example, participant race and ethnicity are often not reported in brain imaging studies, which ignores the potential impact that sociodemographic factors may have on cognitive, biological, or psychological constructs. At the same time, variables pertaining to race and ethnicity, particularly in the context of structural and functional neuroimaging, should not be misinterpreted to underlie race-based biological differences that perpetuate scientific racism (see Section IV.A.iv. on developing mindful theoretical models). Paternalism manifests as making decisions regarding study design and procedures without understanding the experiences of those who are impacted by the decisions and their autonomy. For Black research participants and patients, scholars have examined the historical underpinning of fearing the Black body in the U.S., rooted in a past where an individual’s physical characteristics were utilized to justify the denial of freedom [16]. These ideas did not completely fade with time, and repercussions of this ideology permeate today throughout multiple healthcare systems [17,18]. CTN practitioners must be careful not to perpetuate “colorblind ideologies” by ignoring that racial differences have a contextual foundation that is vital to the knowledge of the field.

CTN also struggles to recognize, reflect, and acknowledge current and past wrongdoings. Examples of harm include perpetuating the notion of race as a biological reality, engaging in racially exclusionary practices, and focusing on individual rather than structural racism [6,17,19]. In general, the vast majority of CTN research has been completed with white heterosexual and able-bodied men, and sometimes women [17]. An important barrier that hinders the ability of neuroscience research to impact and serve actual communities is not understanding the harm associated with denying problematic research practices that disadvantage or inadvertently exclude marginalized individuals.

### Case studies

Enduring challenges in CTN research stem from the tradition of research that is done for, about, or with participants in mind, but not with participants as collaborators in experimental design, data collection, analysis, interpretation, and writing, as has become increasingly important in other disciplines (Community-Based Participatory Research; e.g., [20]). Below, we highlight three specific examples of research methods, practices, and norms that may hinder the translation of neuroscience to mental health clinics: economic disadvantages in procuring neuroscience technologies, use of so-called standardized stimulus sets with non-representative norms, and applications of predictive and normative modeling with fMRI data.

#### Economic disadvantages

CTN research assumes a certain level of economic privilege to participate in experimental treatments involving neuroscience technologies [21]. For example, research has focused on making fMRI useful for precision psychiatry, both in predicting treatment response [22] and augmenting the effects of therapy, e.g., with real-time fMRI neurofeedback [23]. Precision psychiatry is an emerging area in neuroscience and medicine that considers individual characteristics to maximize the effectiveness of treatment and interventions. Bringing this work into the community has been met with consistent challenges across insurance agency and clinician stakeholders, such as the availability of relevant hardware (research scans are frequently acquired on 3 T scanners – whether these results will generalize to the 1.5 T scanners more often available clinically is unclear), the ambiguity regarding who would pay for these uses of fMRI (often ∼$500/hour), and the lack of normative data stratified by variables such as race, age, and gender. Other nominally more available technologies are characterized by inherent biases that prevent ethical clinical translation. For example, because electroencephalography (EEG) is difficult to collect in dense hair, the vast majority of EEG data have been collected from white people (only 5/81 examined EEG articles reported having any black people in their samples; [24]); available EEG-based technologies, which might be used for precision medicine or intervention are not even available to communities with denser hair [24–26]. EEG hardware costs range considerably, with higher end systems that yield better quality data costing ∼$80,000, again, creating barriers for implementation in disadvantaged communities. Similarly, the tools of psychophysiology, such as electrodermal activity, have long been known to vary with or perform poorly with darker skin (e.g., [27]). There are differences, by race, in many baseline psychophysiological measures (e.g., [28]) whereas norms in psychophysiology are generally for white samples, yielding biases in interpretation. Exclusion criteria common to neuroimaging and psychophysiology studies disproportionately rule out minority and disadvantaged samples, based on increased prevalence in marginalized individuals (e.g., head trauma, blood pressure medicines, drug dependence). Indeed, numerous studies have excluded or diminished the importance of racially minoritized participants because the data were believed to be possibly not usable due to different culture norms or that there is a deficit-based explanation for racialized differences in findings [6,29]. For example, biases have been well-documented in biomedical optics CTN research: paradoxically, skin tone bias in diagnostics and neuroimaging has been ignored on the one hand. Black participants have been disproportionately excluded from skin conductance data in fear conditioning studies on the other [18].

#### Diversity versus stimulus set standardization

Another fundamental barrier to effective translation of neuroscience to practice lies within the stimulus sets that are frequently employed in neuroscience paradigms. Exclusion of ethnically minoritized people is acutely obvious in studies that use facial displays of affect or affective scenes as stimuli in cognitive neuroscience paradigms paired with a neuroscience tool, such as fMRI or EEG. With recent exceptions (e.g., [30,31]), representations of facial affect typically employ stimuli sets comprised of white faces. In some cases, this homogeneity is explicitly acknowledged as a methodological decision (i.e., researchers may argue that this exclusion serves to reduce variability in stimuli). However, homogeneity in stimuli is increasingly recognized as a limitation and a threat to generalizability [32]. Neural response to faces can vary based on higher-order perceptions of identity, emotion, similarity, and trustworthiness. These functions are influenced by the social environment, including social experiences of racism, sexism, etc. and individual-level cultural norms. White-standardized stimulus sets may thus be classified as more foreign, novel, or threatening to non-White individuals. These may have a significant effect on data analysis, results, and interpretation [33]. Because of the ubiquity of racially homogenous stimuli sets, this barrier permeates a range of translational neuroscience methods. A growing consensus among neuroscientists and related fields, however, recognizes the significance of this issue across paradigms such as neuroimaging [19], computerized cognitive training [34], early childhood assessment [35], and as discussed below, have called for the adoption of more representative and diverse sets of facial affect stimuli.

Beyond depictions of white faces, a potentially more insidious issue with stimuli used in neuroscience paradigms involves linguistic stimuli, such as sentences, phrases, or single words used in many cognitive neuroscience paradigms, or cognitive training paradigms such as interpretation bias modification. Many such stimuli sets are normed within convenience samples of college students in North America, the majority of whom are white [36]. Few studies have explicitly examined how intersecting factors of race, ethnicity, class, gender, or sexual orientation may influence ratings for such stimuli, yet the ubiquity of college student samples and the limited diversity of such samples raise the significant possibility that translation of paradigms using such verbal stimuli is inherently limited in their potential for uptake in more diverse settings. One study examined racial bias in stimulus sets used in multiple research studies, largely finding that corpi of affective stimulus sets tend to have photos rated, and thus selected, as “negative” which include more ethnically minoritized people, but photos that are rated and used as exemplars of “positive” content include more apparently white people. Explicitly including racially diverse content across valences may require going outside standard normed picture corpi. Racial bias in using standardized stimulus sets is problematic, given well-known demand characteristics associated with responding to nonrepresentative researchers and stimuli and, indeed, studies have shown that researchers who are not diverse tend to propagate perspectives that do not account for diversity [37] and may introduce demand characteristics based on their own positionality (e.g., [38]).

#### Limitations of predictive and normative modeling

A major obstacle to progress in precision psychiatry is that systematic algorithmic biases in current machine learning approaches are overly tuned to majority populations and often fail to generalize to minority populations. For example, predictive models formed on neuroimaging data collected from white participants break down in their performances when used to predict phenotypes for Black participants [39]. Another statistical approach increasingly applied in precision psychiatry, called normative modeling, maps variation in quantitative brain metrics associated with psychiatric conditions. Normative models employ a strategy similar to growth charts in pediatric medicine, where an individual child’s physical measurements are compared to a reference population, and significant deviations may indicate a medical concern. Similarly, in the sibling discipline of neuropsychology, individual cognitive performance is compared to normative reference standards that include faulty separate sets of norms for different racial groups [30]. These have led to calls for removing race as a factor in normative modeling [40], and for taking extra care in determining when a neuropsychological construct can be expected to generalize (i.e., is influenced by race-related psychosocial factors), and when it should not [41]. In CTN, normative models may be used to identify the extent of neural variation of an individual diagnosed with a clinical condition relative to a normative (nondiagnosed) population [42]. However, similar to growth charts and neuropsychological reference norms, such approaches have inherent flaws. First, the ecological validity of the reference group must be considered. When reference groups fail to include individuals from diverse sociodemographic perspectives in the normative population to which individuals are compared, healthy individual differences can be conflated with pathology and further disadvantage marginalized groups. This concern has been discussed by some of the groups who are leading the field of normative modeling [43], but not yet functionally addressed. Second, it remains unclear what percentage or standard deviation difference from a normative model is cause for concern and whether this varies by sociodemographic measurements. Third, the clinical importance of observed differences remains unclear and is subject to clinician bias. If an individual’s pattern of neural variation differs from a normative model, who determines it is cause for concern, and what clinical workup follows is not defined objectively through well-established clinical guidelines. Without objective strategies for clinical decision-making, the known microaggressions and discrimination in clinical bias can impact implementation.

## Mismatch with clinician needs

Research has shown, using computational linguistics, that patients use different vocabulary, reflecting different clinical concerns, than are represented by either treatment providers or institutions that control resources [44]. For example, whereas translational scientists may want to target mechanisms of disorder, patients may want more functional days at work. It is thus possible that scientists could be successful in their research goals and patients’ needs will not have been met. Particularly, there is little data suggesting that the needs of diverse communities are accounted for in translational science, as considerations like systematic racism, which are of particular interest at the community level, are often hard to operationalize or neglected in translational contexts.

A gap between what clinicians perceive as their need to use the results of neuroscience research in their practices and the work being done by translational researchers was highlighted by Strege et al. (2021) [45] who reported that 91% of those surveyed want multiple randomized controlled trials to validate methods before they are adopted as empirically supported treatments. 77% want better prediction of outcomes with representative – which we interpret as diverse – clinical samples. These requirements are at odds with the majority of CTN research that remains focused on small-scale conceptual studies that are often not randomized or which potentially advance to the level of randomized efficacy trials with highly selected samples, but rarely to effectiveness trials, which would satisfy the needs of clinicians. Similarly, 41% of clinicians surveyed requested that translational methods be endorsed by a psychological society, which can be seen as representing endorsement by their chosen community and highlights the importance of researchers to build trust with communities of people who are using and stand to benefit from translational research.

## Serving diverse and marginalized populations

In the next section, we bring attention to two theoretical frameworks (structural competency and adaptive calibration), as well as a research framework (community-based participatory research (CBPR) methods) for CTN.

### Structural competency and social determinants of health

Structural competency models were first conceptualized by Metzl & Hansen (2014) [9] as “the trained ability to discern how a host of issues defined clinically as symptoms, attitudes, or diseases (e.g., depression, hypertension, obesity, smoking, medication “non-compliance,” trauma, psychosis) also represent the downstream implications of a number of upstream decisions about such matters as health care and food delivery systems, zoning laws, urban and rural infrastructures, medicalization, or even about the very definitions of illness and health” (p.126). At the core of this model is a recognition that social, political, and economic forces may produce symptoms or alter neurodevelopment and biology through DNA methylation or other processes. Such forces are sometimes referred to as social determinants of health, which refer to a broad range of factors that are not distributed evenly across the population and contribute to health inequities [46]. The structural competency framework is particularly important in clinically focused disciplines, as it emphasizes systems-level and structural barriers to health, compared to individual-level factors, that are not just the product of interpersonal encounters or biased health providers, but which are caused by structural inequities, and have implications for interventions to address bias and stigma on a policy level. This framework is also important because it recognizes a link between societal processes and the biological variables we measure in research that may be a function of the laws and policies governing access to transportation, housing, wealth, and health. There have already been efforts to integrate structural competency models into medical and psychology training programs [47], including guidelines for developing competencies for working with individuals identifying as lesbian, gay, bisexual, transgender, gender nonconforming, or born with differences in sex development [48].

### Adaptive calibration theory

Adaptive calibration models rooted in evolutionary developmental theories of stress and propose that individuals living in highly stressful environments make strategic resource tradeoffs to adapt and survive [17,49]. Adaptive calibration conceptually differs from deficit-focused models, which may conceive of constructs such as hypervigilance to threat as a deficit that needs to be normalized. More adaptive aspects of surviving systems of oppression are not adequately represented or studied in the neuroscience literature or in research paradigms aimed at understanding dimensions of psychopathology, such as the Research Domain Criteria (RDoC) or Hierarchical Taxonomy of Psychopathology (HiTOP). Adaptive calibration models therefore allow the reconceptualization of certain processes as strengths, rather than deficits, that support resilience and survival. Within the context of stress responsivity, an important implication of this framework is revising conceptualizations of threat and safety based on one’s broader context.

### Community-based participatory research

CBPR [20] refers to an approach to community-engaged research, which involves engaging community members as partners in the design, conduct, and interpretation of research. Although limited community-engaged research has been conducted in translational neuroscience, this is a growing area, and there are already strong examples of integrating CBPR and neuroscientific methods to better serve specific community mental health needs. For example, researchers have collaborated with Indigenous communities to examine cultural identification using multimodal assessments, which necessitates working closely with tribal sovereignty and research regulatory infrastructure to minimize harms and maximize benefits [50]. Integrating CBPR methods in CTN centers the ethics of research and clinical practice within communities and maximizes the potential for findings to be meaningful.

## Bringing Bioethical and Critical Consciousness in CTN into Action

Ethics are complex, contextually driven principles, but ethics alone are not enough. In this section, we outline a curriculum of bioethical and critical consciousness in the form of ten steps that can positively offer added translational impact to CTN research (see Table 1).

**Table 1.** Bringing bioethical and critical consciousness in CTN into action

Target Objective	Approach
Recognizing ethics errors of the past	Cultivate humility by anticipating and proactively addressing bias in research and clinical procedures.
Reducing structural barriers to mental health	Translate knowledge of structural barriers and social determinants of mental health into research practices by providing more support for transportation or meals for research participants, and improving policies for recruiting and retaining marginalized populations, such as by modifying timing of study visits.
Embracing community-based research	Involve community stakeholders in research as partners by soliciting input and feedback on research design and throughout studies as they unfold process.
Embodying mindful theoretical models	Resist the attribution of biological causes to differences between socially defined groups, and utilize alternative study designs that do not simply compare marginalized versus non-marginalized groups.
Incorporating multimodal assessments of lived experiences	Incorporate personalized and ecologically valid stimuli in research studies, such as experiences with discrimination, racial stress, or early life adversity, to acknowledge and measure social experiences that may modulate biological processes.
Utilizing meaningful stimulus sets	Utilize stimulus sets, such as the Racially Diverse Affective Expression Face Stimuli Set (RADIATE [27]), that are representative of the general population.
Innovating with measurement procedures	Develop recruitment materials to address physiologic variability of participants, including skin tone and hair density.
Minimizing bias in predictive models	Conduct research to understand the impact of sociodemographic factors on cognitive and mental health phenotypes in predictive models that rely on machine learning models.
Promoting diversity in the workforce	Support inclusivity of researchers in training, hiring and retention.
Fostering neuroscience literacy	Enhance understanding of the malleability of biological processes and neuroscience research through efforts to improve literacy among clinicians, patients, and the public.

## Actionable solutions to bridge the translational gap

### Understand ethics mistakes of the past

Despite the good intentions of individual researchers and clinicians to uphold the principle of beneficence, ethical missteps in the past highlight how aspirations may not always correspond to actions and ethical values are not universally applied. One example of a major violation of bioethics is the case of Henrietta Lacks, who was a Black woman in 1951 suffering from an aggressive form of cervical cancer, and whose cells were used and shared without her consent [51]. Mistrust is born out of a legacy of neuroscience, medicine, and psychology that developed problematic practices, such as phrenology, eugenics, and intelligence testing as a justification for racial superiority and Black slavery [52]. Because of such historical wrongs, scientists and clinicians alike need to acknowledge that there may be a deep and valid mistrust of our mental health clinic by marginalized people. Given that modern neuroscience-informed interventions rely on medicalized procedures such as with noninvasive brain stimulation or fMRI, a critical consciousness lens that accounts for the experiences of marginalized communities with such technologies is even more important. Ethically conscious scientists and clinicians can proactively address mistrust of biomedical methods by anticipating bias at the outset of the work and engaging with communities.

#### Understand structural barriers to mental health

The structural competency framework associated with critical consciousness fosters an understanding of systems-level factors that can perpetuate social oppression and mental health disparities [9]. Systems-level determinants of mental health may include sociodemographic factors, housing insecurity, transportation availability, and neighborhood factors, such as poverty and unemployment [53]. Structural competency is an extension of cultural competency and implies that the scope of understanding cultural influences on health outcomes must be expanded to the systems level to understand policy-related patterns that shape differential access to mental health resources and interventions. Social barriers to receiving neuroscience-informed interventions must be addressed to succeed in making clinically significant improvements on a population scale. For individual research studies, understanding such structural barriers may translate to shifting the timing and access of research study visits to accommodate those who may need more support to attend visits (e.g., extending study visits beyond typical business hours, providing transportation or meal vouchers for longer visits). At an institutional level, researchers can work with their departmental units and local IRBs to shape recruitment and retention practices and improve equitable access to research for marginalized groups.

#### Integrate CBPR approaches and build trust

CBPR can serve as a guiding framework for conducting inclusive CTN. “Nothing about us without us” is a simple and strong guiding principle. CBPR approaches enhance the broad reach of science and reduce health disparities for marginalized groups in the process but does not apply only to health equity research. It encourages collaboration with individuals and groups outside of academia, and attention to participants as decision-makers in the research process. These can be family members, caregivers, teachers and other educators, paraprofessionals, spiritual leaders, and policymakers. Not having to remove street clothes during fMRI scans, being able to access and understand their own data, or hearing about incidental findings that allow them to take clinical action, may open opportunities for modifying study procedures are some examples of how input can shape study procedures. Involving additional stakeholders helps to develop a shared language and build trust in the research process, in the value of neuroscience in informing psychological and physical health, and in developing a common understanding of the causes of mental health disorders. Scientists who lack prior experience examining health disparities should be cognizant of quick solutions that may fail them in the long term. Sustainable, proactive solutions will come from an acquired understanding of health equity work that has already been conducted with communities of interest. To avoid erasure of historical efforts, community partners should be acknowledged on a timeline and in a manner that is beneficial to them. This may not necessarily lead to a metric such as authorship in a peer-reviewed manuscript that is only unidirectionally meaningful if results are not further shared in outreach or given back to those – individuals and communities – who offered their primary data.

#### Develop mindful theoretical models

One major challenge in neuroscience is how researchers use socially defined groups in analyses and then hypothesize a biological – rather than social – basis for any observed group differences. One example of this is in research comparing people by sexual orientation. While significant research has shown very limited evidence for a biological basis for sexual orientation [54], comparing neural structure and function between heterosexual and nonheterosexual people has been an ongoing topic of interest in neuroscience (for systematic review and critique, see [55]). However, recent research has shown that it is the impact of differential social experiences (i.e., victimization) and not orientation itself that influences differences in neural functioning [56]. Other examples of biological embedding have emerged in the threat processing literature [29], which underscores the bi-directional relationship between life experiences and biological processes.

It is critical for CTN to explore research strategies that understand how biology interacts with the differential social experiences that come with marginalization to produce mental health disparities [57]. To do this effectively, researchers need to be mindful of methods for (1) collecting and reporting sociodemographic information on people; (2) optimizing community engagement to inform research questions and analyses; (3) interpreting results that neither overinterpret findings towards social categorizations as a function of biology nor ignoring important group differences that could aid understanding of disparities; and (4) translating results equitably to reduce suffering in the communities disproportionately impacted [58].

The most effective research study designs may therefore not translate to group comparisons between marginalized and nonmarginalized groups, as marginalized communities may be valuable to study in their own right without reference to comparative populations. Health equity and developmental psychopathology researchers have long advocated for study designs that focus on within-person differences over time, recognizing that relying solely on cross-sectional, between-group comparisons may obscure important variations in lived experiences and clinical presentations [29,59–63]. The use of between-group designs should involve careful consideration of how metrics from marginalized populations are compared with those from majority groups to avoid reinforcing inequities by perpetuating the notion of the majority experience as the standard.

#### Use multimodal assessments of lived experiences

Laboratory-based paradigms designed to capture biopsychosocial influences on health inequities will benefit from the use of personalized and ecologically valid stimuli that capture the unique challenges of minority stress (e.g., laboratory analogs) [64,65]. Studies may also include measures of life experiences that theoretically modulate the biological processes or interventions of interest, such as measures of early adverse life experiences, traumatic experiences, or experiences with everyday racism and discrimination. However, there are limitations to the validity, acceptability, and clinical utility of screening measures for adverse childhood experiences, as well as barriers to implementing trauma-informed care in clinical research settings [66,67]. Paradigm development should involve incorporating feedback from community experts and insights gleaned from qualitative research.

#### Utilize diverse stimulus sets

Despite the historical reliance on stimuli that reflect white faces or stimuli normed among predominantly white samples, recent commentaries and reviews have encouraged researchers to seek out and utilize stimulus sets that reflect greater racial diversity [19,34,35]. An example of such a database is the Racially Diverse Affective Expression Face Stimuli Set (RADIATE [30]). Unfortunately, there are few comparable examples of verbal stimuli developed and normed by racially diverse participant pools; developing these stimuli will be a positive accomplishment for future research.

#### Adopt inclusive psychophysiological methods

Psychophysiological methods such as EEG and electrodermal activity are linked to the unintended exclusion of Black participants from translational neuroscience research [64,65]. Although there is increasing emphasis on the introduction of novel hardware to address these disparities, there are immediate steps that researchers can take to improve measurement with existing systems [24,25,68]. For example, informed by CBPR, scholars now recommend more inclusive and equitable recruitment materials that directly acknowledge concerns regarding the potential impact of EEG on hair with dense coils, particularly for people with hairstyles requiring substantial time and financial upkeep. Additionally, scheduling research appointments flexibly to accommodate changes in hairstyles can increase inclusion. Moreover, adopting the 15-20 minute braiding technique to increase electrode contact with the scalp can be used with existing systems [25]. For instance, as part of the EEG Hair Project, researchers at the Biomechanics, Rehabilitation, and Interdisciplinary Neuroscience (BRaIN) Lab at the University of Central Florida enlisted the expertise of a local Black hairstylist to train research staff in this braiding method, aiming to promote greater equity and inclusion for Black participants [69].

#### Identify and mitigate bias in predictive models

There is an urgent need to develop a more complete understanding of the role that sociodemographic factors characterizing aspects of population diversity (e.g., participant gender, race, ethnicity, and income) play in cognitive and mental health phenotype prediction [69]. Identification and explicit modeling of the major sources of population stratification is a necessary milestone towards developing justice, equity, diversity, and inclusion (JEDI)-informed machine learning frameworks. This can be done by constructing predictive modeling solutions that jointly acknowledge a broad portfolio of interindividual differences and is a necessary precondition for the responsible use of population neuroscience data to avoid further disadvantaging underrepresented groups and communities. For example, systematic bias in who gets classified as carrying a particular medical or mental health diagnosis could reinforce disparities in access to health care and services. Underdeveloped practices in predictive modeling and inappropriate handling of demographic stratification can lead to further exclusion and stigmatization of groups that have historically been marginalized in healthcare institutions. Since the reproducibility, generalizability, and clinical utility of published machine learning models have rarely been put to the test in broadly sampled, more diverse cohorts, deepening our understanding of the full extent and consequences of generalization failure in neuroscience is a critical paradigm shift necessary for developing a truly JEDI-informed precision psychiatry that serves the diverse global population. It is also necessary to frame results and questions carefully, especially when considering participant race and ethnicity, to counteract harmful biases [70] and improve health-related predictions at the single-subject level. Documenting the extent of neural variation along with diverse sociodemographic factors may be an important step for making stronger inferences about variation that falls within healthy and pathological ranges, so long as large and diverse datasets can be examined.

#### Recruit and retain a diverse research workforce

The likelihood that translational insights will be applied beyond well-resourced majority populations will be improved when marginalized communities are represented not just as community partners, but as mental health scientists in their own right Governmental agencies, funding bodies, and other key players in the decision-making process of who enters and advances down the pipeline from trainee to principal investigator have started to attend more to JEDI issues than before. Yet the leakiness of this pipeline, especially for trainees from marginalized communities, is well documented [71,72]. Inclusivity of scientific ecosystems at all levels of training will be improved when these factors come into sustainable focus [73].

#### Provide training in neuroscience literacy

Supporting the development of neuroscience literacy among clinicians is another important step toward the adoption of neuroscience in mental health clinics in marginalized communities. Clinicians may be hesitant to adopt neuroscience-informed tools in the clinic because they are unfamiliar with neuroscience terminology and because they may not have received training in the tools neuroscience has to offer [74]. In addition, a training emphasis on CBPR-informed neuroscience studies may also increase the uptake of these tools by emphasizing education modules most likely to positively impact care for marginalized communities. It is important to consider not only doctoral-level clinicians but especially the much greater number of mental health workers at the master’s level who conduct trauma work in marginalized communities. Development of neuroscience literacy can be supported by offering free Continuing Education credits across mental health professions to educate clinicians about how neuroscience can be applied in the clinic, as well as integrating a neuroscience curriculum into training programs. Enhancing neuroscience literacy may also be beneficial for patients and research participants. For example, information on how brain and disorder mechanisms change with treatment, sometimes referred to as neuroeducation, may directly impact clinical care by supporting treatment [75], increase patient openness to participating in neuroscience studies.

## Conclusion

Neuroscience research involving biological variables and interventions will most beneficially impact clinical practice for mental health disorders if it contends with biases that are revealed and mitigated through a combined bioethical and critical consciousness framework approach. We outline actionable steps to maximize the potential of the approach. These steps are informed by structural competency, adaptive calibration, and community-based participation in research. By pairing critical consciousness with bioethical consciousness in CTN, we believe that significant strides will be made in achieving enduring relief from the burdens of mental illness.
